# Differences in Abortion Rates between Asian Populations by Country of Origin and Nativity Status in New York City, 2011–2015

**DOI:** 10.3390/ijerph18126182

**Published:** 2021-06-08

**Authors:** Sheila Desai, Mary Huynh, Heidi E. Jones

**Affiliations:** 1 Guttmacher Institute, New York, NY 10038, USA; 2 New York City Department of Health and Mental Hygiene, New York, NY 10013, USA; mhuynh@health.nyc.gov; 3 Graduate School of Public Health and Health Policy, City University of New York, New York, NY 10027, USA; heidi.jones@sph.cuny.edu

**Keywords:** abortion, Asians, New York City

## Abstract

Despite the size of the Asian population in New York City (NYC) and the city’s robust abortion surveillance system, abortion-related estimates for this population have not been calculated previously. This study examined the use of abortion services among specific Asian groups in NYC from 2011–2015. Using NYC surveillance data, we estimated abortion rates for Asians, disaggregated by five country of origin groups and nativity status, and for other major racial/ethnic groups. We compared rates between groups and over time. From 2014–2015, the abortion rate for Asian women in NYC was 12.6 abortions per 1000 women aged 15–44 years, lower than the rates for other major racial/ethnic groups. Among country of origin groups, Indian women had the highest rate (30.5 abortions per 1000 women), followed by Japanese women (17.0), Vietnamese women (13.0), Chinese women (8.8), and Korean women (5.1). Rates were higher for U.S.-born Asian groups compared to foreign-born groups, although the differential varied by country of origin. The abortion rate declined or remained steady for nearly all Asian groups from 2011–2015. These findings reinforce the importance of disaggregating data on this population at multiple levels and begin to provide much-needed evidence on the use of abortion services among Asian groups.

## 1. Introduction

One in four women aged 15–44 years in the United States (U.S.) will have an abortion in her lifetime; it is a common experience in the U.S. and a critical component of sexual and reproductive health care [1]. Understanding socio-demographic patterns of abortions in the U.S. provides important context to identify how policies, service-related barriers, or other structural inequities may differentially shape access to abortion for specific groups. Indeed, robust abortion surveillance can be one public health strategy to ensure equitable access to and delivery of abortion services across all populations [2].

Although abortion data and patterns of service use have been examined by the major racial/ethnic groups in the U.S., Asian groups are typically excluded from these studies because of sample size limitations. As a result, little is known about Asian women’s use of abortion care, though they are nearly 10% of the female reproductive-age (15–44 years) population in the country [3]. Specifically, data on the prevalence of abortion, as estimated by the abortion rate, are rarely calculated for Asian populations in the United States. Asians comprise over 50 ethnicities with large variations in national origin and nearly 70% are foreign-born [3,4]. Due to differences in immigration patterns and the impact of colonialism on some Asian countries, health behaviors and outcomes between Asian groups may differ substantially. Yet, abortion-specific measures remain non-existent by country of origin or nativity status, although other types of health service use have been shown to differ by these factors [5]. Even at a local level, these data are rare. For example, New York City (NYC), with nearly 15% of the population identifying as Asian, and the vast majority (80%) of whom are immigrants, represents the largest Asian population in any U.S. city [6]. Yet, abortion-related estimates for Asian groups in NYC have not been calculated previously, obscuring the use of abortion services among these populations and in comparison to other groups. Without such evidence, we risk ignoring critical reproductive health needs of Asian groups and upholding the harmful “model minority” myth that Asians are a universally privileged group, whose health needs do not require attention [7].

Research indicates that a notable share of Asians are uninsured, experience linguistic isolation, and do not receive comprehensive health services [8,9]. For example, studies have found that Asian women are less likely than non-Hispanic White women to receive critical reproductive health services such as prenatal care, breast and cervical cancer screening and management, and sexually transmitted infection testing and treatment [10,11]. Furthermore, over one-third of the Asian population has limited English proficiency, indicating a need for multilingual health services [9]. Cultural values within some Asian groups may also discourage open conversations related to reproductive and sexual health care, limiting information-seeking or service utilization [12]. Combined with mounting legal and logistical barriers to obtaining abortion [2] and immigration policies that restrict health coverage for many immigrants [13], these factors could contribute to differential use of abortion care not only between Asian and White women, but also within the Asian population and between immigrant and non-immigrant Asians.

In this study, we examine use of abortion services among Asian groups in NYC. New York State (and City) has relatively expansive access to abortion compared to other parts of the United States. For example, abortion is legal in New York up to 24 weeks gestation (and in subsequent weeks if there are health risks associated with continuing the pregnancy or the fetus is not viable) and the state requires abortion coverage in private and public health insurance plans. Within this context, we use surveillance data from Asian groups in NYC to calculate abortion rates for Asians overall and by country of origin and nativity status, compare rates between groups, and examine changes in rates over time by group. With this analysis, we begin to identify group-specific patterns in service use and delivery of abortion in previously understudied groups. These data can inform local and broader efforts to support Asian groups’ access to fundamental reproductive health services.

## 2. Materials and Methods

### 2.1. Data Sources

Aggregate-level counts of abortions reported between 2011 and 2015 were provided by the NYC Department of Health and Mental Hygiene (DOHMH), Bureau of Vital Statistics. Abortion counts include in-clinic abortions up to 24 weeks gestation and medication abortions up to 10 weeks (where medications are dispensed by facilities). These data came from “Induced Termination of Pregnancy” reports, completed by health care providers, who are required by the municipal health code to report all surgical and medication abortions performed in NYC, regardless of individual residency status, to the DOHMH. Data were provided by race/ethnicity, country of origin, and nativity status. To avoid small cell sizes and preserve confidentiality, counts were provided in pooled years: 2011–2013 and 2014–2015. These data represent a census of facility-based abortions in NYC occurring from 2011–2015.

NYC population data were obtained from the American Community Survey (ACS). The Integrated Public-Use Microdata Series (IPUMS)-USA database, provided by the University of Minnesota, was used to obtain 1% samples of the ACS for each year from 2011–2015 [14]. Annual data were pooled to mirror the year intervals of the NYC data. NYC population distributions by age, race/ethnicity, and nativity were estimated using weighted tabulations of the ACS; these distributions were applied to the total number of NYC women aged 15–44 years, obtained from the NYC DOHMH vital statistics reports, to estimate population counts by these characteristics.

In accordance with institutional Human Research Protection Program procedures, this research was considered exempt from IRB review given its use of existing and de-identified aggregate-level data.

### 2.2. Measures

We calculated group-specific abortion rates by dividing the number of abortions in a specific group by the number of female NYC residents aged 15–44 years in that same group; we then multiplied this figure by 1000. The abortion rate reflects use of abortion services and can indicate how likely abortion is in a particular group. We calculated the abortion rate by the following self-reported descriptors:

Race/ethnicity: Group-specific abortion rates were calculated for non-Hispanic Asian, non-Hispanic White, non-Hispanic Black, and Hispanic populations in NYC. Asians include individuals who reported having origins in the Far East, Southeast Asia, or the Indian subcontinent.

Country of origin: Among Asians, the abortion rate was further disaggregated by country of origin and calculated for Indian, Chinese, Japanese, Korean, and Vietnamese women. Membership to these groups is defined by where individuals (or their families) were born. They represent the five largest Asian country of origin groups in NYC, although they are not inclusive of all Asians in NYC.

Nativity status: For these five groups and Asians overall, the rate was also calculated for foreign-born individuals (i.e., immigrants) and U.S.-born individuals (i.e., non-immigrants).

Finally, although we use the term “women” to describe population groups throughout this paper, many trans men, gender non-binary, and gender non-conforming people also need and have abortions.

### 2.3. Analyses

We calculated 2014–2015 abortion rates by race/ethnicity and the five Asian country of origin groups. Nativity-specific rates were then calculated for Asians overall and for each Asian country of origin. Finally, group-specific rates were calculated for 2011–2013 and the percent change between 2011–2013 and 2014–2015 was estimated. We chose 2011–2013 as our point of comparison given changes in data collection beginning in 2011 that provided more specificity in race/ethnicity and country of origin groups for Asians [15].

The incidence of pregnancy and abortion varies considerably by age, so we age-standardized abortion rates to account for differences in the underlying age composition between the groups under study. The rates were age-standardized to the 2011–2013 NYC population of women, given minimal changes to this population overall and within racial/ethnic groups between 2011 and 2015 (see Table S1 for population weights). Counts for this age-standardization reference population were compiled from the annual Summary of Vital Statistics reports prepared by the NYC DOHMH, Bureau of Vital Statistics.

## 3. Results

### 3.1. Comparing Abortion Rates between Asians and Other Major Racial/Ethnic Groups

The 2014–2015 abortion rate for Asian women in NYC was 12.6 abortions per 1000 women. This rate was lower than the rates for all other major racial/ethnic groups (15.7 per 1000 for non-Hispanic White women; 63.5 per 1000 for non-Hispanic Black women; and 33.9 per 1000 for Hispanic women) as well as the overall average (Table 1).

### 3.2. Comparing Rates between Asian Country of Origin Groups

When we disaggregated Asian women by their country of origin, we found that Indian women had the highest abortion rate (30.5 per 1000 women) followed by Japanese and Vietnamese women (17.0 and 13.0 per 1000 women, respectively). The abortion rates for Chinese and Korean women (8.8 and 5.1 per 1000, respectively) were lower (Table 1). Notably, each of these rates also varied substantially from the abortion rate for Asian women overall.

### 3.3. Comparing Rates between Immigrants and Non-Immigrants within Asian Groups

The abortion rate for U.S.-born Asian women was approximately 1.5 times higher than for foreign-born Asian women. The direction of this relationship by nativity status persisted within every country of origin group, although the magnitude of difference varied. For example, the abortion rate for U.S.-born Chinese women was nearly twice as high as that for foreign-born women, while among Japanese women, we found a nearly five-fold difference in the abortion rate between foreign-born (8.4 abortions per 1000 women) and U.S.-born women (38.9 per 1000) (Table 2).

### 3.4. Examining Changes in the Abortion Rate between 2011–2013 and 2014–2015

Table 3 presents the percent change in abortion rates between 2011–2013 and 2014–2015 by race/ethnicity and by nativity within Asian country of origin groups. The total NYC abortion rate declined by 13% during this time period. The rate remained steady for Asian and non-Hispanic White women, and declined by 16% for non-Hispanic Black women and by 17% for Hispanic women. The abortion rate among foreign-born Indian women declined 8% during this time period, while a modestly greater decline (11%) was observed among their U.S.-born counterparts. We found similar declines by nativity for Chinese and Japanese women. Although we also documented declines in the rates for Korean women over this time, the percent change was greater for foreign-born (42%) compared to U.S.-born women (22%). The rate among all Vietnamese women increased, although the magnitude of the change was greater among foreign-born (91%) compared to U.S.-born (9%) women.

## 4. Discussion

In 2014–2015, the abortion rate differed between Asians and other major racial/ethnic groups and within Asian country of origin groups in NYC, reinforcing the importance of disaggregated data for Asian populations. The abortion rate for Asian women overall was 2.5 to five times lower than the rate for Hispanic and non-Hispanic Black women. Examining the data by country of origin, we found that the abortion rate among Indian women was nearly two to six times higher than the rates among the other groups. When data on Asians were further disaggregated by nativity status, the abortion rate was higher for U.S.-born women compared to immigrant women in all groups. Finally, we found that the abortion rates among Asian, non-Hispanic Black, and Hispanic women in NYC remained steady or declined between 2011–2013 and 2014–2015, reflecting national- and state-level changes in this measure [16,17]. With the exception of Vietnamese women, we saw declines in the abortion rates among all country of origin groups, regardless of nativity.

Differences in fertility patterns between groups may contribute to some of this variation in abortion rates. Indeed, higher abortion rates have been attributed to higher unintended pregnancy rates and a greater percentage of unintended pregnancies resolving in abortion [18]. Although we do not have data on unintended pregnancies among Asian women in NYC, we know that this population had the lowest pregnancy rate in 2015 (82.6 pregnancies per 1000 women) compared to the rates of other major racial/ethnic groups (119.6, 98.0, and 88.6 for non-Hispanic Black, Hispanic, and non-Hispanic White women, respectively). During this time, Asian women in NYC also had the highest birth rate at 16.6 births per 1000 women, followed by 14.7 among non-Hispanic White women, 14.3 among Hispanic women, and 12.1 among non-Hispanic Black women [19]. Asian women’s low prevalence of pregnancies and high prevalence of births relative to other racial/ethnic groups in NYC could underlie the lower abortion rate observed among this population in this study.

Documented differences in abortion rates between country of origin groups may also be attributed to similar variations in pregnancy rates, although further investigation is needed as these data are not readily available for Asian groups. It is also possible that the ability to obtain abortions differs between Asian groups, especially if access to health coverage, logistic and financial resources, and supportive social networks—factors documented to impact abortion access [20,21,22]—vary between subgroups. For example, 2015 data from the NYC Community Health Survey suggest that Korean and Chinese New Yorkers—the two country of origin groups with the lowest abortion rates among Asians in this study—are more likely than other Asian groups to be uninsured and have lower English proficiency, which could complicate their access to care [23].

These barriers to care may also be amplified among immigrants in these groups, impacting their use of or access to sexual and reproductive health services, including abortion. Indeed, that all Asian immigrant groups had lower abortion rates than their U.S.-born counterparts may signal obstacles to health care that they, like other immigrants in NYC, face such as limited provider outreach or knowledge of care and coverage options. Especially given limited English proficiency (LEP) in particular Asian immigrant groups, ensuring access to high-quality medical interpretation services or, more optimally, language-concordant care could facilitate patient–provider communication, trust, and patient-centered care, as demonstrated among other LEP groups [24]. Expanding the capacity of the NYC health care system to provide culturally competent and linguistically diverse health services is essential to facilitating accessible and comprehensive health care, including abortion [25]. It is also possible that specific Asian groups hold sociocultural norms and beliefs that stigmatize pregnancy decisions, especially abortion, for some women, hindering their health-seeking behaviors or attitudes [12]. Finally, the differences by nativity status within Asian groups may be related to migration effects on fertility, which could lead to the postponement of childbearing until after migration [26]. Prior research has suggested that some immigrant women, depending on their age at migration, have higher fertility compared to their non-immigrant counterparts [27]; especially if these pregnancies are more likely to be intended, immigrants’ use of abortion services may be lower than non-immigrants’, as found in this analysis. That said, this study does not examine age at migration or fertility desires and further research is needed to assess the influence of tempo or migration effects in this population.

The decline in abortion rates for nearly all Asian groups likely reflects the national downward trend in the abortion rate, which has been attributed to changes in contraceptive use [1]. At the same time, the observed declines among Asian immigrants, in particular, might suggest reduced access to care due to barriers potentially related to the immigrant experience, including the changing social and political climate in the United States. Indeed, recent evidence has suggested that ongoing anti-immigrant rhetoric coupled with increasing fear of arrest, detention, and deportation of recent years may increasingly deter immigrants as well as mixed-status families from obtaining needed health care, potentially including abortion, even in sanctuary cities such as NYC [28,29,30]. However, more research is needed to understand the dynamics behind these trends over time.

This study has several limitations. First, the abortions included in this analysis are likely undercounts. Recent findings from the Guttmacher Institute indicate that the NYC DOHMH abortion reporting system identified 85% of facility-based (or facility-initiated) abortions performed in the city [31]. In addition to those procedures missed by the municipal surveillance system, abortions occurring outside of health facilities (e.g., a hospital, clinic, or physician’s office) may be underreported in these data. Although the magnitude of this underreporting is likely to be relatively small [32,33], underreporting of abortions could be higher in Asian or immigrant estimates if these groups are more likely than their counterparts to obtain abortions in informal settings or self-manage their abortions. In that case, the abortion rates calculated in this study for these groups may be underestimated. Given potential concerns around reporting immigration status, immigrants may have been more likely than non-immigrants to provide inaccurate information regarding their nativity. As a result, we might expect the abortion rates among U.S.-born women to be slightly inflated. Furthermore, patterns in abortion use observed in this study may not be generalizable to Asian populations living in other areas of the country, particularly those that are more restrictive. NYC has a high density of abortion providers and does not have any of the major abortion restrictions (e.g., waiting periods or limitations on health insurance coverage) found in other states [17]. Finally, given restricted availability, the surveillance data used in this study do not reflect the most recent annual counts of abortions or include individual-level data related to each abortion. As current data become more available, further study of abortion rates among these populations and underlying factors that may inform differentials will be important. Despite these limitations, these data offer novel insights to understand the use of abortion services among Asian groups in this country.

## 5. Conclusions

This study improves our understanding of abortion prevalence among specific Asian groups in NYC and serves as a scientific anchor for future research and policies that seek to advance the reproductive health of Asian American populations. While the abortion rates in this study cannot tell us about people’s lives or experiences obtaining services, they do demonstrate that abortion is common and necessary for all groups, including Asians. We will continue to need robust evidence and data on abortion in Asian groups to identify appropriate and effective approaches to safeguard access to these services.

Furthermore, comprehensive and granular data are integral to understanding public health trends, including trends in abortion access and use, and this study underscores the value and feasibility of examining abortion data disaggregated at multiple levels. Our findings provide important baseline data to identify future changes in the use of abortion services in these groups in NYC. Compiling and updating this evidence remains particularly critical in the current political context, in which efforts to restrict abortion may impose a significant burden on both women of color and immigrant women seeking abortion care [34,35]. With documented chilling effects in health care use across immigrant communities and families, future research and surveillance efforts that continue to evaluate abortion use across Asian groups in NYC and the country, more broadly, are indicated.

This study should also serve as a launch pad for future similar research in other cities with sizeable Asian populations, such as Vancouver, Metro Toronto, London, or San Francisco. A more robust and geographically diverse body of evidence on abortion rates among the Asian diaspora could help illuminate how different abortion and immigration landscapes inform variations in use of services. Such information could ultimately help researchers, public health professionals, and policymakers better serve and support the reproductive health needs of diverse Asian populations—within and outside of NYC—seeking abortion care.

Finally, bringing into focus data on abortion from Asian and immigrant women, populations that often go uncounted in reproductive health research and policy, not only helps to center and prioritize their experiences, but also contributes to dismantling harmful racial and cultural myths about these groups. Collectively, this information will be essential to inform effective public health programs and policies that ensure access to comprehensive reproductive health care for all.

## Figures and Tables

**Table 1:** Age-standardized abortion rates among New York City women by race, ethnicity, and country of origin: 2014–2015.

Race/Ethnicity	Population Counts	Abortion Rate per 1000 Women
All women	3,881,904	33.8
Asian	590,099	12.6
Non-Hispanic White	1,196,373	15.7
Non-Hispanic Black	846,089	63.5
Hispanic	1,139,653	33.9
Asian subgroups by country of origin		
Indian	115,907	30.5
Chinese	261,097	8.8
Japanese	18,056	17.0
Korean	54,043	5.1
Vietnamese	6485	13.0

**Table 2:** Age-standardized abortion rates among Asian populations in New York City by nativity status, 2014–2015.

Race/Ethnicity and Nativity	Population Counts	Abortion Rate per 1000 Women
Asian		
Foreign-born	431,947	10.6
U.S.-born	158,153	15.7
Asians disaggregated		
Indian		
Foreign-born	84,867	26.8
U.S.-born	31,040	35.9
Chinese		
Foreign-born	188,666	7.0
U.S.-born	72,432	12.1
Japanese		
Foreign-born	14,363	8.4
U.S.-born	3693	38.9
Korean		
Foreign-born	36,654	2.3
U.S.-born	17,389	8.9
Vietnamese		
Foreign-born	4834	8.9
U.S.-born	1650	14.9

**Table 3:** Abortion rates and percentage change in rates between 2011–2013 and 2014–2015 among New York City women by race/ethnicity, country of origin, and nativity status.

Race/Ethnicity, Country of Origin, and Nativity Status	Abortion Rate per 1000 Women		Percent Change
	2011–2013	2014–2015	
All women	39.0	33.8	−13
Asian	12.2	12.6	3
Non-Hispanic White	15.7	15.7	0
Non-Hispanic Black	75.8	63.5	−16
Hispanic	40.6	33.9	−17
Asian groups by nativity status			
Indian			
Foreign-born	29.1	26.8	−8
U.S.-born	40.4	35.9	−11
Chinese			
Foreign-born	7.4	7.0	−5
U.S.-born	13.2	12.1	−9
Japanese			
Foreign-born	9.1	8.4	−8
U.S.-born	45.1	38.9	−14
Korean			
Foreign-born	4.0	2.3	−42
U.S.-born	11.4	8.9	−22
Vietnamese			
Foreign-born	4.7	8.9	91
U.S.-born	13.6	14.9	9

## Data Availability

Data were obtained from the New York City Department of Health and Mental Hygiene, Vital Statistics Bureau.

## Supplementary Materials

The following is available online at https://www.mdpi.com/article/10.3390/ijerph18126182/s1, Table S1: New York City reference population counts and weights for age standardization: Women ages 15–44 years, 2011–2015.

## References

[1] Jones R.K., Jerman J. (2017). Population group abortion rates and lifetime incidence of abortion: United States, 2008–2014. Am. J. Public Health.

[2] American Public Health Association (2015). Restricted Access to Abortion Violates Human Rights, Precludes Reproductive Justice, and Demands Public Health Intervention. https://www.apha.org/policies-and-advocacy/public-health-policy-statements/policy-database/2016/01/04/11/24/restricted-access-to-abortion-violates-human-rights.

[3] US Census Bureau (2015). Asian Alone or in Combination with One or More Other Races: 2015 American Community Survey 1-Year Estimates. https://factfinder.census.gov/faces/tableservices/jsf/pages/productview.xhtml?pid=ACS_15_1YR_B02011&prodType=table.

[4] National Council of Asian Pacific Americans (2013). Best Practices: Researching Asian Americans, Native Hawaiians and Pacific Islanders.

[5] Tapales A., Douglas-Hall A., Whitehead H. (2018). The sexual and reproductive health of foreign-born women in the United States. Contraception.

[6] US Census Bureau’s American Community Survey Office (2013). American Community Survey, 2009–2013. http://factfinder2.census.gov.

[7] Yi V., Museus S. (2015). Model Minority Myth. The Wiley Blackwell Encyclopedia of Race, Ethnicity, and Nationalism.

[8] Islam N., Trinh-Shevrin C., Rey M. (2009). Introduction: Towards a Contextual Understanding of Asian American Health. Asian American Communities and Health: Context, Research, Policy, and Action.

[9] Ramakrishnan K., Ahmad F. (2014). Language Diversity and English Proficiency.

[10] Ghosh C. (2003). Healthy People 2010 and Asian Americans/Pacific Islanders: Defining a baseline of information. Am. J. Public Health.

[11] Chawla N., Breen N., Liu B., Lee R., Kagawa-Singer M. (2015). Asian American women in California: A pooled analysis of predictors for breast and cervical cancer screening. Am. J. Public Health.

[12] Okazaki S. (2002). Influences of culture on Asian Americans’ sexuality. J. Sex. Res..

[13] Healthcare.gov (2017). Coverage for Lawfully Present Immigrants. https://www.healthcare.gov/immigrants/lawfully-present-immigrants/.

[14] Ruggles S., Flood S., Goeken R., Grover J., Meyer E., Pacas J., Sobek M. (2018). Integrated Public Use Microdata Series: Version 8.0 [Dataset].

[15] Toprani A., Madsen A., Das T., Gambatese M., Greene C., Begier E. (2014). Evaluating New York City’s abortion reporting system: Insights for public health data collection systems. J. Public Health Manag. Pract..

[16] Jones R.K., Jerman J. (2017). Abortion incidence and service availability in the United States, 2014. Perspect. Sex. Reprod. Health.

[17] Guttmacher Institute (2018). State Facts about Abortion: New York.

[18] Jatlaoui T.C., Boutot M.E., Mandel M.G., Whiteman M.K., Ti A., Petersen E., Pazol K. (2018). Abortion Surveillance—United States, 2015. MMWR Surveill. Summ..

[19] Gwynn C., Van Wye G., Betancour F., Borrell J., Huynh M., Mino M., Le E., Li W., Sebek K. (2017). Summary of Vital Statistics 2015, The City of New York. New York City Department of Health and Mental Hygiene, Bureau of Vital Statistics. https://www1.nyc.gov/assets/doh/downloads/pdf/vs/2015sum.pdf.

[20] Jerman J., Frohwirth L., Kavanaugh M.L., Blades N. (2017). Barriers to Abortion Care and Their Consequences for Patients Traveling for Services: Qualitative Findings from Two States. Perspect. Sex. Reprod. Health.

[21] Fuentes L., Lebenkoff S., White K., Gerdts C., Hopkins K., Potter J.E., Grossman D. (2016). Women’s experiences seeking abortion care shortly after the closure of clinics due to a restrictive law in Texas. Contraception.

[22] Jones R.K., Upadhyay U.D., Weitz T.A. (2013). At What Cost? Payment for Abortion Care by U.S. Women. Women’s Health Issues.

[23] King L., Deng W. (2018). Health Disparities among Asian New Yorkers. New York City Department of Health and Mental Hygiene. https://www1.nyc.gov/assets/doh/downloads/pdf/epi/databrief100.pdf.

[24] Parker M.M., Fernández A., Moffet H.H., Grant R.W., Torreblanca A., Karter A.J. (2017). Association of Patient-Physician Language Concordance and Glycemic Control for Limited-English Proficiency Latinos with Type 2 Diabetes. JAMA Intern. Med..

[25] Flores G. (2006). Language Barriers to Health Care in the United States. N. Engl. J. Med..

[26] Wilson B. (2020). Understanding How Immigrant Fertility Differentials Vary over the Reproductive Life Course. Eur. J. Popul..

[27] Tønnessen M., Wilson B. (2020). Visualising Immigrant Fertility Profiles of Childbearing and their Implications for Migration Research. Int. Migr. Integr..

[28] Watson T. (2014). Inside the refrigerator: Immigration enforcement and chilling effects in Medicaid participation. Am. Econ. J. Econ. Policy.

[29] Page K.R., Polk S. (2017). Chilling Effect? Post-Election Health Care Use by Undocumented and Mixed-Status Families. N. Engl. J. Med..

[30] Rodriguez R.M., Torres J.R., Sun J., Alter H., Ornelas C., Cruz M., Fraimow-Wong L., Aleman A., Lovato L.M., Wong A. (2019). Declared impact of the US President’s statements and campaign statements on Latino populations’ perceptions of safety and emergency care access. PLoS ONE.

[31] Jones R.K. (2019). Personal Communication.

[32] Grossman D., Holt K., Peña M., Lara D., Veatch M., Córdova D., Gold M., Winikoff B., Blanchard K. (2010). Self-induction of abortion among women in the United States. Reprod. Health Matters.

[33] Jones R.K. (2011). How commonly do US abortion patients report attempts to self-induce?. Am. J. Obstet. Gynecol..

[34] Hasstedt K., Boonstra H. (2018). Taking Stock of Year One of the Trump Administration’s Harmful Agenda Against Reproductive Health and Rights. Guttmacher Institute. https://www.guttmacher.org/article/2018/01/taking-stock-year-one-trump-administrations-harmful-agenda-against-reproductive.

[35] Howell M., Starrs A. (2017). For Women of Color, Access to Vital Health Services is Threatened. Guttmacher Institute. https://www.guttmacher.org/article/2017/07/women-color-access-vital-health-services-threatened.

