# Supplementary material for: Differences in Abortion Rates between Asian Populations by Country of Origin and Nativity Status in New York City, 2011–2015
Source: Int J Environ Res Public Health. 2021 Jun 8;18(12):6182. doi: 10.3390/ijerph18126182 (PMC8229373; doi:10.3390/ijerph18126182)
Supplement: Supplementary file 1 [file ijerph-18-06182-s001.zip › ijerph-1215026-supplementary.pdf]

International Journal of
Environmental Research and Public Health

Supplementary Material

Table S1. New York City reference population counts and weights for age standardization: Women ages 15–44 years, 2011–2015.

Age Group	2011–2013 [1]		2014–2015	
	**Population Count**	**Weight**	**Population Count**	**Weight**
15–19 yrs	733,654	0.127517	467,786	0.120504
20–29 yrs	2,158,457	0.375164	1,452,722	0.374229
30–39 yrs	1,978,512	0.343887	1,376,611	0.354623
40–44 yrs	882,748	0.153431	584,785	0.150644
Total	5,753,371	1	3,881,904	1

[1] The 2011–2013 population and weights were used as the reference for age standardization.
